# Recent Advances in High-Performance Bioinspired Sustainable Materials for Automotive Applications

**DOI:** 10.3390/ma19183884

**Published:** 2026-09-11

**Authors:** Kanchan Kumari, Swastik Pradhan, Monalin Mishra, Abhishek Barua, Chitrasen Samantra, Trilochan Rout, Manisha Priyadarshini

**Affiliations:** 1 Department of Mechanical Engineering, Parala Maharaja Engineering College, Berhampur 761003, Odisha, India; kanchan.1087@gmail.com (K.K.); chitramech4u@gmail.com (C.S.); trilochan.me@pmec.ac.in (T.R.); 2 School of Mechanical Engineering, Lovely Professional University, Phagwara 144411, Punjab, India; swastik.rock002@gmail.com; 3 Environment & Sustainability Department, CSIR–Institute of Minerals and Materials Technology, Bhubaneswar 751012, Odisha, India; monalinmishra.1996@gmail.com; 4 Department of Automobile Engineering, Parala Maharaja Engineering College, Berhampur 761003, Odisha, India; 5 Academy of Scientific and Innovative Research (AcSIR), Ghaziabad 201002, Uttar Pradesh, India; 6 CGU BEST Centre, C V Raman Global University, Bhubaneswar 752054, Odisha, India; any.manisha@yahoo.com.au

**Keywords:** bioinspired materials, sustainable materials, automotive engineering, biomimetic composites, lightweight structural materials, green manufacturing

## Abstract

Electrified mobility regulations and lifecycle emissions targets have increased the demand for lightweight structural materials in vehicle architectures. Bioinspired composite materials offer microstructural configurations that alter conventional trade-offs among specific stiffness, crash energy absorption, and manufacturing energy requirements. This review evaluates the translation of biological structural archetypes including nacre, bamboo, cortical bone, and lotus leaves into load-bearing and functional automotive components. Quantitative benchmarks of continuous natural-fiber laminates, bio-cellular lattices, and mycelium-based acoustic cores are compared against high-strength steel and aluminum alloys. Key mechanical and functional metrics, including specific energy absorption (ranging from 35 to 48 kJ kg^−1^ for bioinspired crash structures), dynamic loss factors, and Cassie-Baxter superhydrophobic surface stability, are evaluated alongside high-throughput manufacturing routes such as high-pressure resin transfer molding (HP-RTM) and additive manufacturing. Methodological parameters for ISO 14040/14044-compliant Life Cycle Assessment (LCA) are synthesized, emphasizing component-level functional units over gravimetric mass equivalence. Furthermore, operational boundaries, specifically hygrothermal interfacial degradation, matrix glass transitions (T_g_ < 120 °C), and multi-axial loading sensitivity, are systematically outlined to define design limits for automotive deployment.

## 1. Introduction

The automotive sector is experiencing a radical change in response to ever more stringent environmental laws, lofty carbon-neutrality goals, and an increasing demand for energy-efficient means of transportation. The global push for low-carbon mobility and sustainable production, including the European Union (EU) Green Deal, the Fit for 55 legislative package (targeting a 55% net greenhouse gas reduction by 2030), and increasingly stringent Corporate Average Fuel Economy (CAFE) standards, has driven this transition. Concurrently, the proliferation of electric vehicles (EVs) has shifted regulatory and engineering focus from tailpipe emissions to complete life-cycle impacts spanning raw-material extraction, service durability, and end-of-life recovery. Material selection therefore determines a large share of a vehicle’s life-cycle environmental impact [1,2].

The lightweight engineering principle is a key factor in achieving high fuel efficiency in vehicles since the reduction in structural weight has a direct effect on fuel consumption in traditional internal combustion engine vehicles and on the range of battery electric vehicles. Most EV batteries weigh several hundred kg, and lightweight structural components must be designed that do not compromise the integrity or crashworthiness of the vehicle. Advanced high-strength steels (AHSS), aluminum alloys, magnesium alloys, carbon-fiber-reinforced polymers (CFRPs), etc., are some of the conventional lightweight materials that have played a significant role in vehicle lightweighting. However, their production is usually energy-intensive, has large greenhouse gas emissions, uses limited availability of minerals and, in many instances, is not recyclable. These constraints have posed great challenges to the circular and sustainable automotive industry [1,2,3].

Hierarchical biological architectures achieve mechanical efficiency by coordinating stress distribution, crack deflection, and strain energy dissipation across multiple length scales. Natural structures evolve under multi-parameter physiological constraints (e.g., metabolic energy limits, ambient synthesis, and growth kinetics) to achieve resilient, multi-objective trade-offs rather than maximizing single mechanical properties [4,5]. Translating these balanced design strategies into engineered composites yields lightweight architectures with superior damage tolerance [4,6,7].

**Figure 1:**
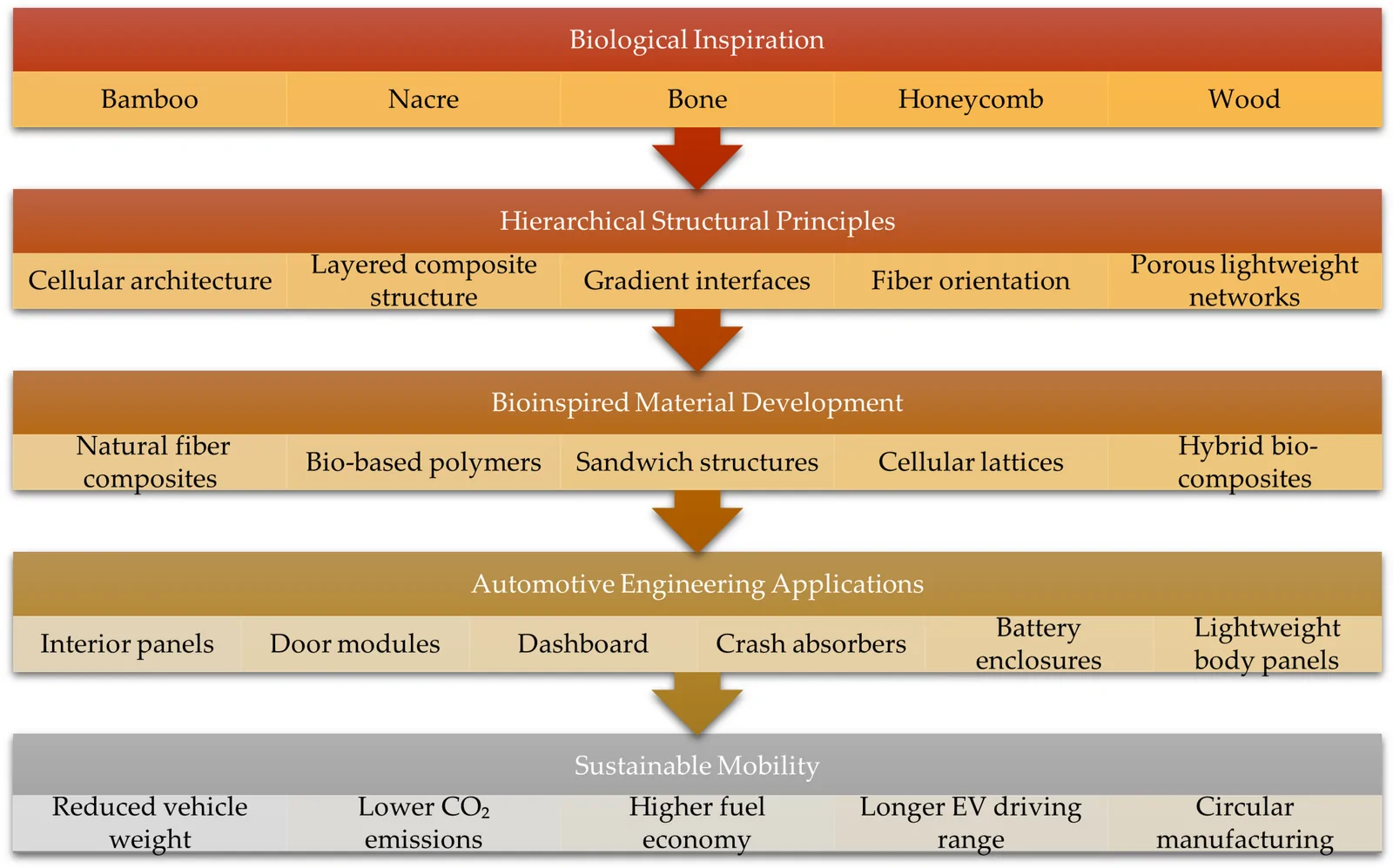
Conceptual Framework of Bioinspired Sustainable Materials for Automotive Engineering.

Bioinspired vehicular materials can be said to have passed through three stages of development. The main materials used in the first generation were natural fibers without any treatment, which were used as fillers in polymer compounds, resulting in environmental benefits but with limited structural properties. Second generation materials featured chemical surface treatments and better fiber-matrix interfaces to provide increased strength, durability and water resistance. In recent years, third generation bioinspired materials have combined renewable fibers, bio-based polymers, advanced manufacturing processes, computational optimization, and biological inspired hierarchical architectures to create multifunctional lightweight materials for structural and semi-structured automotive parts [1,3,4,8]. Figure 1 shows the conceptual framework that represents the translation of biological design principles into high-performance bioinspired materials for sustainable automotive engineering [1,4,5,8].

**Figure 2:**
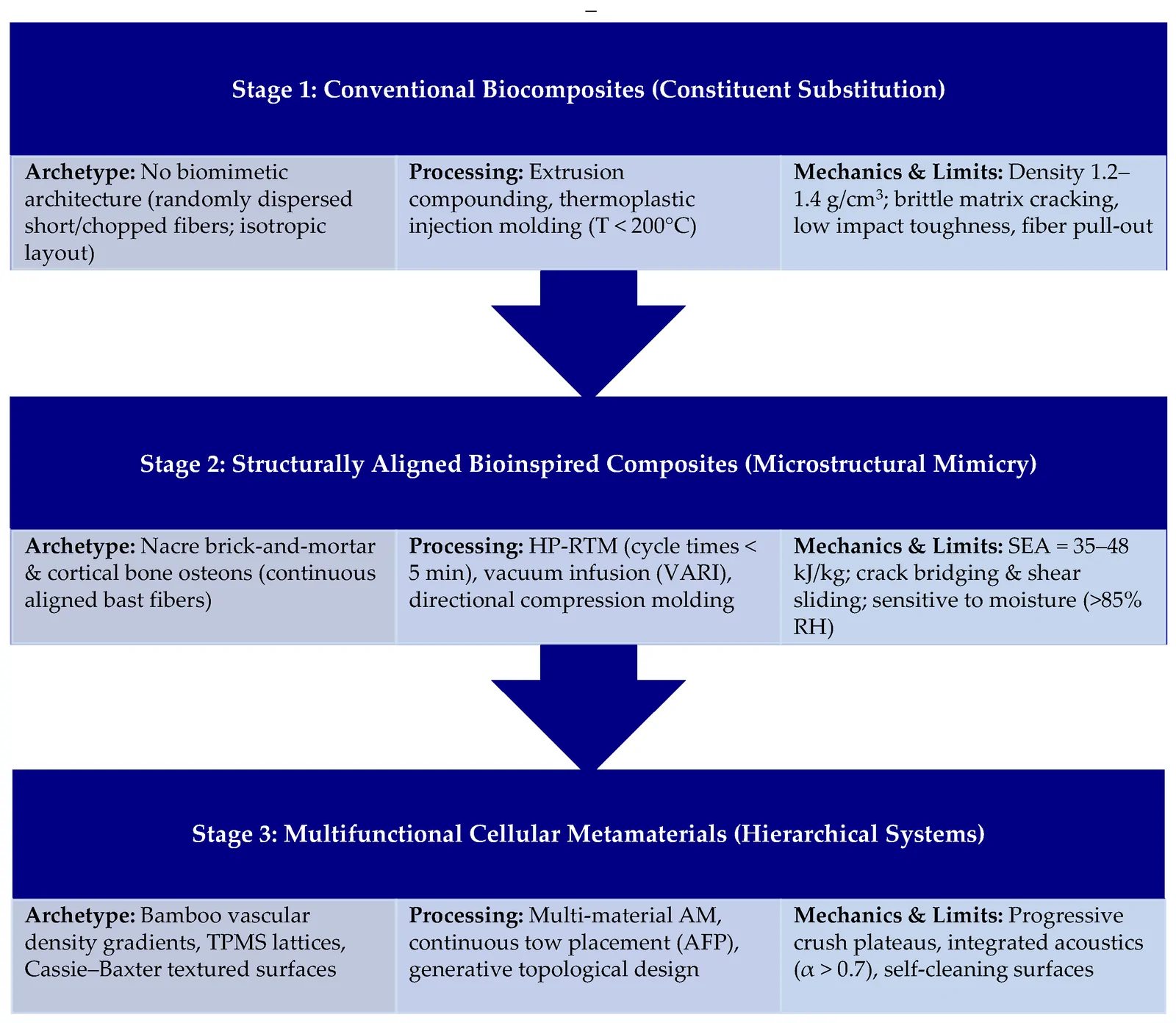
Classification matrix of bio-based and bioinspired materials across structural archetype, fabrication method, and mechanical response.

Carbon-neutral mobility has completely changed the material selection strategies in the automotive sector. Previous lightweighting efforts were mainly directed at operational fuel usage, but many design philosophies are now focused on minimising life cycle environmental effects, such as the production of materials and their manufacturing, use, recycling and disposal. This transition has led to a greater focus on the development of lightweight materials with high specific strength, superior impact resistance, low embodied carbon, and compatibility with circular production systems. The use of sustainable bio-based materials is therefore an important alternative to the traditional engineering materials for the development of future vehicle platforms [5,6,9,10,11]. The progressive transition from non-architected biocomposites to microstructurally aligned bioinspired laminates and multifunctional cellular metamaterials across distinct manufacturing and mechanical performance domains is illustrated in Figure 2 [1,4,7,8]. Table 1 shows a comparison between conventional automotive materials and the bioinspired sustainable materials, their biological inspirations and representative applications.

Owing to progressive microstructural failure mechanisms (such as fiber debonding, platelet sliding, and cellular buckling), bioinspired composites exhibit stable energy absorption under dynamic crash loads, mitigating abrupt catastrophic failure. Renewable natural fibres such as flax, hemp, kenaf, bamboo, jute, and sisal generally have lower embodied energy and carbon footprints than conventional reinforcements, while bio-based polymer matrices can support recycling, biodegradation, and circular manufacturing [1,2,3,8,9,10]. These characteristics have encouraged their use in interior trim panels, dashboards, door modules, battery enclosures, crash-energy absorbers, acoustic insulation, and semi-structural components. Nevertheless, industrial automotive deployment is governed by critical hygrothermal, thermal, and interfacial stability limits during service, as well as high-rate processing constraints [9,10]. Resolving these technical barriers demands the rigorous coupling of biological design strategies with advanced composite manufacturing and empirical life-cycle engineering.

To synthesize state-of-the-art developments, a literature screening was conducted across Web of Science, Scopus, and IEEE Xplore databases for peer-reviewed studies published up to 2026. Search queries utilized combinations of keywords including bioinspired, biomimetic, natural-fiber composite, crashworthiness, specific energy absorption, and life cycle assessment. Retrieved studies were evaluated based on reported mechanical metrics (such as specific stiffness, specific energy absorption (SEA), and loss factor), manufacturing scalability, and environmental indicators modeled under ISO 14040/14044 frameworks [3].

This review covers developments in bioinspired materials for automotive applications reported up to 2026. Some of the biological principles of bioinspired material design, renewable reinforcement systems, agricultural and food-waste particulate fillers, mycelium foam cores, bio-based matrices, processing technologies, hierarchical structural architectures, mechanical performance, automotive applications, life-cycle sustainability, and commercialization prospects are discussed. The emphasis is placed on understanding and explaining the links between biological inspiration and hierarchical microstructure, structural performance and environmental sustainability, giving a scientific basis for the development of next generation lightweight materials for sustainable mobility.

## 2. Fundamentals of Bioinspired Sustainable Materials

To establish an unambiguous basis for automotive material selection, key classifications must be distinguished:

- Bio-based materials are defined by their renewable feedstock origin (e.g., natural plant fibers, bio-epoxies, and fungal mycelium), irrespective of whether their internal microstructure emulates nature.
- Bioinspired materials incorporate architecture, gradients, or functional mechanisms abstracted from biological models, such as nacre-like staggered platelets or bamboo-like graded tubes, fabricated from either bio-based or synthetic constituents.
- Biomimetic materials represent a specialized subset replicating exact physical mechanisms or interface morphologies, such as the Cassie–Baxter superhydrophobic papillae of lotus leaves.
- Sustainable materials require empirical life-cycle validation (via ISO 14040/14044 LCA) demonstrating measurable environmental benefits, including lower cumulative energy demand and reduced global warming potential across cradle-to-grave boundaries.

### 2.1. Principles of Bioinspiration in Materials Design

Bioinspired materials are developed by abstracting the structural, functional, and adaptive mechanisms of living systems to create high-performance engineering components. While synthetic materials frequently demand high temperatures, elevated pressures, and energy-intensive chemical refinement, biological systems synthesize resilient architectures via bottom-up molecular and cellular self-assembly at ambient temperature and pressure (T ≈ 20–37 °C, P ≈ 1 atm) [6,7]. This metabolic resource efficiency has motivated the development of sustainable composites that balance structural performance with reduced environmental burdens [6,7,11].

Bioinspiration extends beyond copying external macroscopic shapes; it requires identifying the underlying micromechanical mechanisms responsible for biological functionality and translating them into quantitative engineering rules. The design philosophy of biomimetic automotive materials can be categorized into five core principles [4,6,7,12]:

- Hierarchical multi-scale organization, arranging structural features across nano- to macro-scales to facilitate progressive load transfer and crack bridging.
- Evolutionary multi-parameter optimization, which reconciles conflicting structural demands (e.g., load-bearing capacity versus compliance and metabolic expenditure) rather than isolating single peak properties.
- Resource-efficient load-path alignment, channeling material strictly along primary stress trajectories (analogous to trabecular bone remodeling) to eliminate redundant mass.
- Multifunctional integration, embedding secondary capabilities such as acoustic damping, thermal dissipation, or passive self-cleaning directly into the primary load frame.
- Functionally graded transitions, using spatial density or compositional gradients to mitigate interfacial stress concentrations.

### 2.2. Classification of Bioinspired Sustainable Materials

Based on the biological systems that inspire them and the engineering functions they perform, bioinspired sustainable materials can be classified into five major categories: structural bioinspired composites, cellular and architected lattice structures, functional bioinspired surfaces, autonomous self-X and adaptive systems, and supramolecular self-assembled nanomaterials [6,7,11,12,13].

#### 2.2.1. Structural Bioinspired Composites

Structural bioinspired composites emulate the multi-scale organization of load-bearing biological tissues to increase fracture toughness without incurring excessive mass penalties. In nacre, approximately 95 vol.% aragonite platelets are assembled within a thin viscoelastic biopolymer interlayer. This staggered brick-and-mortar geometry suppresses catastrophic crack propagation via progressive interfacial shear sliding, crack bridging, mineral-bridge pull-out, and tortuous deflection paths. Similarly, cortical bone dissipates fracture energy through concentric osteon lamellae and compliant cement lines that twist and arrest propagating microcracks, while wood utilizes aligned tubular cellulose fibrils embedded in a lignin/hemicellulose matrix to sustain high axial bending stresses [5,6,7,11].

#### 2.2.2. Cellular and Lattice Bioinspired Structures

Cellular and architected lattice structures optimize material placement to sustain flexural loads and absorb compressive impact energy at minimal relative density. Bamboo achieves bending stiffness via a radial gradient of vascular bundles, concentrating denser reinforcing fibers along the outer cortex where bending stresses are maximal. Emulating these spatial density gradients in engineered tubular crash boxes, Voronoi topologies, and triply periodic minimal surface (TPMS) lattices prevents sudden global plastic buckling, yielding stable plateau stress regimes under dynamic crushing loads [7,14].

#### 2.2.3. Functional Bioinspired Surfaces

Functional bioinspired surfaces utilize micro- and nanoscale interface topographies to regulate interfacial energy, wettability, and aerodynamic boundary layers. Lotus leaves (Nelumbo nucifera) maintain superhydrophobicity via the Cassie–Baxter state, in which air trapped within dual-scale micro-papillae (10–20 μm) and sub-micron epicuticular wax asperities prevents liquid intrusion, producing static contact angles above 150° and roll-off angles below 5°. Conversely, the aligned longitudinal riblets of fast-swimming sharks modify turbulent boundary-layer vortices to reduce hydrodynamic wall shear stress, while gecko setae exploit van der Waals interactions across billions of nanoscale fibrillar spatulae to produce reversible, residue-free dry adhesion [12,13].

#### 2.2.4. Autonomous Self-X and Adaptive Systems

Autonomous and adaptive material systems incorporate dynamic chemical bonds or responsive reservoirs to detect damage and restore structural continuity. Extrinsic self-healing relies on microcapsules or vascular networks that rupture upon cracking to release polymerizable monomers (such as dicyclopentadiene or epoxy precursors) into fracture planes, recovering up to 90% of localized load-bearing capacity. Intrinsic self-healing exploits reversible covalent networks such as Diels–Alder adducts, disulfide exchange, and transesterification—or non-covalent supramolecular interactions (hydrogen bonding and ionic coordination) to enable repeatable, stimulus-driven crack resealing without depleting active agents [7,13].

#### 2.2.5. Supramolecular and Self-Assembled Nanomaterials

Supramolecular systems leverage non-covalent, reversible molecular interactions including π–π stacking, electrostatic interactions, and metal–ligand coordination to synthesize ordered nanomaterials under ambient processing conditions. Unlike permanent covalent polymer networks, supramolecular interfaces dynamically reconfigure under localized mechanical stress or environmental shifts. When incorporated into composite interphases or barrier nanocoatings, these assemblies improve interfacial shear transfer and suppress microcrack growth while maintaining low processing energy requirements [7,11].

### 2.3. Structure–Property Relationships of Bioinspired Sustainable Materials

The mechanical response of bioinspired sustainable materials is governed by constituent phase fractions, fiber orientation, and interfacial mechanics. To evaluate multi-axial structural performance quantitatively, anisotropic natural-fiber laminates and graded metamaterials require orthotropic constitutive modeling and micromechanical formulations rather than idealized isotropic approximations [6,7,11,12,15].

#### 2.3.1. Hierarchical Structural Organization

Hierarchical architectures influence mechanical behavior through changes in load transfer, stress distribution, interfacial deformation, and failure progression across different structural scales. Rather than introducing additional qualitative descriptions of biological structures, these effects can be incorporated into constitutive and micromechanical models through parameters such as constituent volume fraction, fiber orientation, aspect ratio, porosity, and interfacial strength. Accordingly, the structure–property relationship of bioinspired materials can be evaluated quantitatively using effective elastic properties, specific stiffness, orthotropic compliance, and effective geometric parameters.

#### 2.3.2. Constitutive Relationships for Bioinspired Composites

The mechanical response of bioinspired fiber-reinforced composites is governed by their constituent properties, interfacial load transfer, and multiscale structural orientation. Because natural-fiber architectures exhibit pronounced directionality and orthotropic anisotropy across automotive components, planar elastic deformation under multidirectional service loads is accurately described using the orthotropic compliance tensor:

(1) $$\left[\begin{array}{c} \varepsilon_{1}\\ \varepsilon_{2}\\ \gamma_{12} \end{array}\right]=\left[-\begin{array}{ccc} \frac{1}{E_{1}} & -\frac{\nu_{21}}{E_{2}} & 0\\ \frac{\nu_{12}}{E_{1}} & \frac{1}{E_{2}} & 0\\ 0 & 0 & \frac{1}{G_{12}} \end{array}\right]\left[\begin{array}{c} \sigma_{1}\\ \sigma_{2}\\ \tau_{12} \end{array}\right]$$

where ε_1_, ε_2_, and γ_12_ represent the in-plane normal and shear strains, σ_1_, σ_2_, and τ_12_ denote the corresponding normal and shear stresses E_1_ and E_2_ are the longitudinal and transverse elastic moduli, G_12_ is the in-plane shear modulus, and ν_12_ and ν_21_ are the major and minor Poisson’s ratios, respectively. This constitutive relationship is widely employed to predict the deformation behavior of laminated bio-composites subjected to multidirectional loading [7,15].

#### 2.3.3. Influence of Hierarchical Architecture on Mechanical Performance

Beyond intrinsic constituent moduli, architectural kinematics dictate stress redistribution across staggered and cellular interfaces. In nacre-inspired composites, for example, platelet geometry and interface strength influence the balance between interfacial sliding, platelet pull-out, and direct crack propagation. A commonly used effective platelet aspect ratio is expressed as:

(2) $$S_{eff}=l/t$$

where l and t denote the platelet length and thickness, respectively. The effective aspect ratio, together with interfacial shear strength, controls stress transfer between adjacent platelets and consequently affects the deformation and fracture response of layered bioinspired composites [5,6,7,11]. Higher aspect ratios improve interfacial shear transfer provided that adequate bonding is maintained; however, excessively rigid interfaces suppress pull-out sliding, promoting catastrophic trans-platelet fracture. Similarly, in functionally graded cellular media, continuous spatial variations in relative density and cell wall thickness tailor the crushing stress plateau and prevent localized buckling instabilities under impact (Table 2).

#### 2.3.4. Computational Design and Structure–Property Optimization

Computational engineering provides a systematic approach for optimizing bioinspired architectures before fabrication. Finite element analysis (FEA), multiscale modeling, topology optimization, and machine learning can be used to evaluate the effects of cellular geometry, material distribution, fiber orientation, and interfacial characteristics under prescribed loading conditions. Topology optimization can identify material distributions that maximize stiffness or energy absorption for a specified mass constraint, while finite element simulations can resolve local stress concentrations, deformation patterns, buckling, and progressive failure in complex architectures. Machine learning approaches can further establish relationships between geometric descriptors and target properties, enabling rapid screening of large design spaces. By parameterizing multi-scale unit cells (representative volume elements, RVEs) and interfacial constitutive models, numerical optimization directly predicts non-linear crash deceleration profiles and strain energy dissipation paths prior to physical prototyping, significantly reducing developmental iterations for complex biomimetic architectures [7,13].

## 3. Bioinspired Materials for Automotive Applications

**Figure 3:**
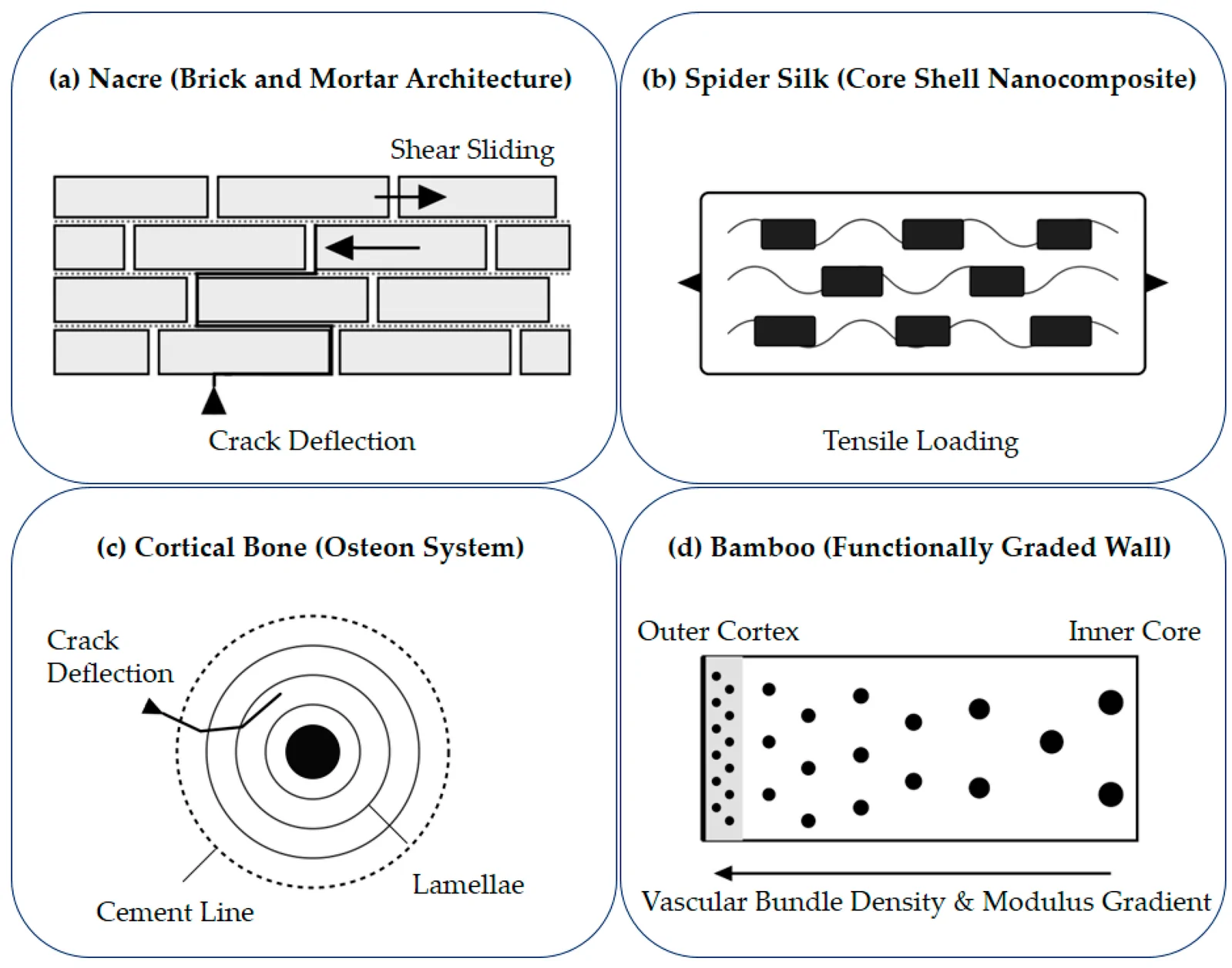
Schematic illustration of four representative biological archetypes, their hierarchical microstructural features, and dominant mechanical deformation mechanisms.

**Figure 4:**
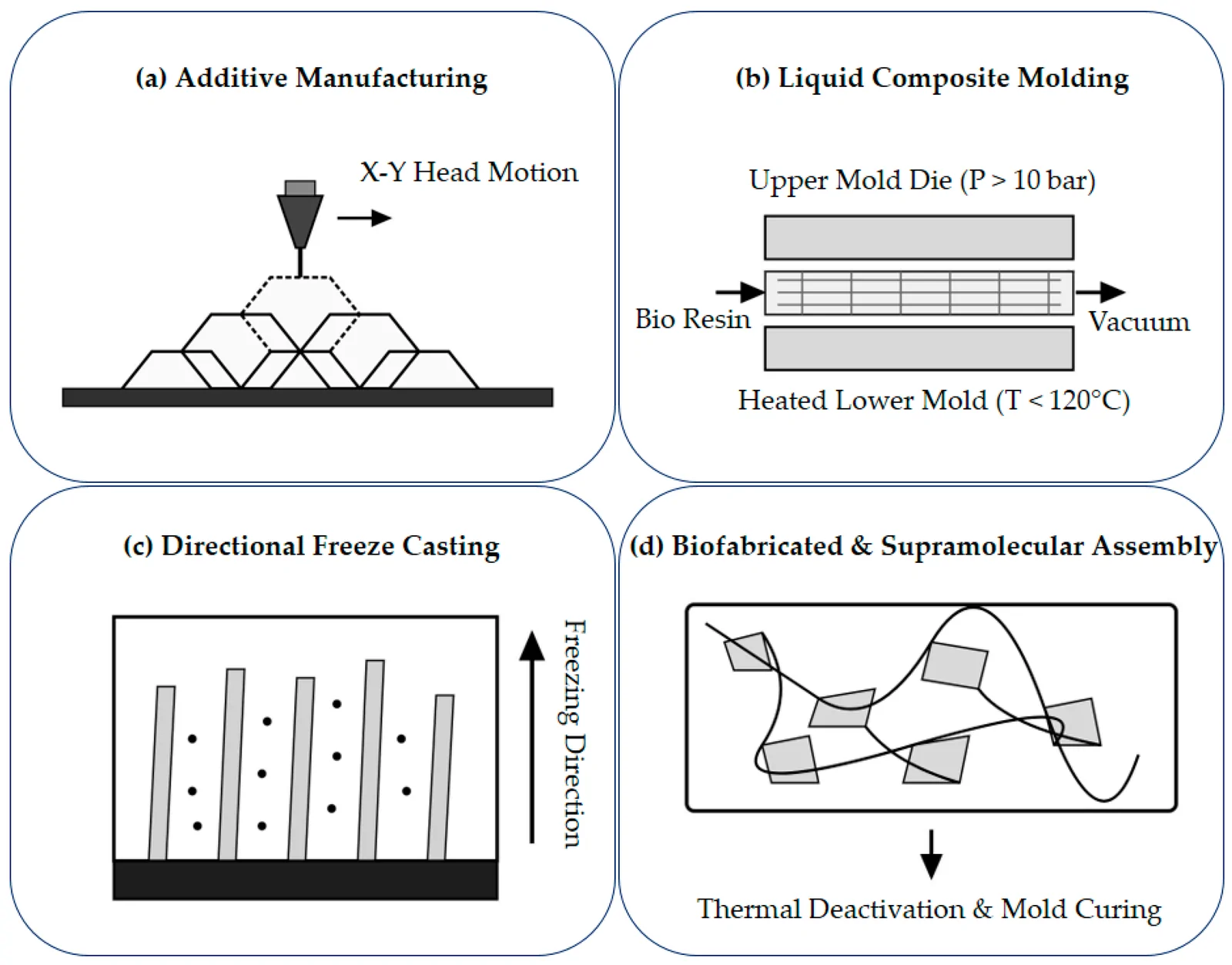
Engineering fabrication and processing pathways for bioinspired automotive materials.

Modern vehicle platforms require engineering materials that simultaneously reconcile conflicting design criteria: maximizing crash-energy absorption and structural rigidity while minimizing mass and life-cycle environmental burdens [1,2,9]. Biological structures such as nacre, bone, and bamboo meet similar trade-offs by combining multi-scale load distribution, strain-energy dissipation, and progressive damage containment (Figure 3) [4,5,6]. As illustrated in Figure 3, four representative biological models illustrate key deformation and strengthening mechanisms: (a) nacre achieves extreme crack resistance through brick-and-mortar platelet sliding, mineral bridge pull-out, and tortuous crack deflection [5,6,11]; (b) spider silk combines crystalline β-sheet nanocrystals with a compliant peptide matrix to deliver entropic elasticity and high tensile strain energy dissipation [7,16]; (c) cortical bone leverages concentric osteon lamellae and cement-line boundaries to promote microcrack arrest, ligament bridging, and fatigue crack shielding [6,7,10]; and (d) bamboo utilizes a functionally graded radial distribution of vascular bundles to optimize flexural rigidity and resist local buckling during bending or compressive crushing [8,9,14]. Translating these biological archetypes into scalable automotive components requires specialized engineering processing pathways capable of replicating hierarchical architectures without inducing thermal degradation in bio-based constituents (Figure 4) [1,15,16]. As shown in Figure 4, modern manufacturing routes encompass four key fabrication domains: (a) additive manufacturing (3D/4D printing) for architected lattice and cellular topologies with spatially tuned porosity [8,14,17]; (b) liquid composite molding (RTM, HP-RTM) for high-throughput continuous natural-fiber preforms with cycle times below 60 s [1,3,15,18]; (c) directional freeze-casting (ice-templating) for producing lamellar and porous anisotropic scaffolds with high compressive energy absorption [19,20,21]; and (d) biofabricated and supramolecular self-assembly for growing low-energy, circular mycelium foam cores and assembling nanostructured interfacial coatings [1,3,11,15]. Unlike simple material substitution, bioinspired engineering couples nature’s microstructural mechanics with scalable manufacturing and computational optimization to produce lightweight, damage-tolerant automotive subsystems with improved lifecycle sustainability [16,17,18,22].

### 3.1. Lightweight Structural Bioinspired Materials

The density of natural fibres commonly used in automotive composites is approximately 1.2–1.5 g cm^−3^, substantially lower than that of steel (7.85 g cm^−3^) and aluminium alloys (2.70 g cm^−3^). This density difference provides a basis for reducing component mass when the required stiffness and strength can be achieved through appropriate fibre volume fraction, orientation, laminate architecture, and matrix selection. Natural fibres such as flax, hemp, kenaf, jute, sisal, and bamboo consist primarily of cellulose microfibrils embedded within lignin- and hemicellulose-rich matrices. Their mechanical response is therefore strongly anisotropic and depends on fibre morphology, microfibril angle, moisture content, fibre–matrix adhesion, and processing conditions [16,22,23]. Surface treatments, including alkali treatment, silane treatment, acetylation, and plasma modification, can alter fibre surface chemistry and improve interfacial bonding while reducing moisture sensitivity. When combined with matrices such as bio-epoxy, PLA, and polyhydroxyalkanoates (PHA), these fibres can be configured into high-performance laminates and core–shell architectures to replace conventional metallic and synthetic composite structural assemblies [16,22,23].

(a) Nacre-Inspired Laminated Composites: Translating the staggered brick-and-mortar deformation mechanisms defined in Section 2.2.1, natural-fiber laminates dissipate dynamic impact energy through progressive fiber debonding, interfacial shear sliding, and controlled interlayer delamination rather than brittle fragmentation. Their crash and impact performance can be quantified using the SEA, defined as
  (3) $$SEA=\frac{E_{abs}}{m}=\frac{\int_{0}^{\delta_{max}}F\left(\delta \right)d\delta }{m}$$
  where E_abs_ is total energy absorbed (kJ), F(δ) is instantaneous crushing force (kN), $\delta_{max}$ is effective crushing stroke (m), and m is specimen mass (kg). Reported SEA values for representative bioinspired and natural-fibre laminates consistently fall within 35–48 kJ kg^−1^ (Table 3), with energy absorption governed by fiber alignment, volume fraction, stacking sequence, and strain rate [10,15,18]. When properly aligned with primary crash paths, these laminated architectures offer specific energy dissipation competitive with metallic crash structures [16,22].
(b) Bamboo-Inspired Cellular Structures: Implementing the radial density gradients described in Section 2.2.2, additively manufactured lattice crash tubes distribute crush loads progressively across vehicle side-impact members. This concept can be translated into engineered structures by varying cell size, strut thickness, porosity, or reinforcement distribution according to the local load requirement. Numerical and experimental studies have investigated Voronoi lattices, honeycomb cores, octet-truss structures, gyroid lattices, and TPMS architectures for controlling stiffness, buckling, peak crushing force, and energy absorption. Depending on relative density and cellular topology, functionally graded bamboo-inspired lattices achieve SEA values ranging from 30 to 45 kJ kg^−1^ (Table 3).

By tailoring the spatial density gradient, progressive plastic folding is promoted across the cellular network, preventing sudden global buckling and stabilizing deceleration pulses during side-impact and front-rail collisions [17,18,22].

### 3.2. Sustainable Bio-Based Fillers, Mycelium Composites, and Circular Biopolymers

In addition to continuous and woven plant fibres, non-structural and semi-structural components also use mycelium composites, agricultural-waste fillers, and recycled or marine-derived biopolymers:

(a) Mycelium-Based Foams and Acoustic Cores: Mycelium composites are biofabricated by inoculating fungal networks (e.g., Ganoderma lucidum, Pleurotus ostreatus) onto agricultural waste substrates such as hemp hurds, sawdust, and cotton gin trash. During growth, the hyphae branch out to bind the organic particles into a coherent, low-density (0.10–0.35 g cm^−3^) porous network. When thermally deactivated, the resulting lightweight core provides an acoustic absorption coefficient (α > 0.7 in the 1000–4000 Hz frequency range) and thermal insulation comparable or superior to expanded polystyrene (EPS) and petroleum-based polyurethane (PU) foams. These acoustic attenuation properties position mycelium-grown panels as functional, fire-resistant replacements for non-structural interior NVH trim modules (Table 4) [1,3,15].
(b) Agricultural Pomace, Fruit Pulp, and Nut Shell Bio-Fillers: Thermoplastic injection molding dominates high-volume interior automotive production for dimensionally stable cabin consoles and structural trim assemblies (Table 4). To replace mineral fillers like talc and calcium carbonate, micronized agro-waste powders derived from almond shells, hazelnut shells, walnut shells, and fruit pomace (e.g., apple and citrus pulp) are integrated into polypropylene (PP), PLA, and polyamide (PA) matrices at 10–40 wt% loadings [1,11,15]. These particulate fillers impart increased flexural modulus and dimensional stability, reduce sink marks in molded components, and lower the net polymer consumption without requiring specialized synthetic chemical modifications [2,11].
(c) Circular Recycled Fibers, Chitin, and Algae-Derived Polymers: Circular vehicle strategies increasingly rely on recycled cotton and wool non-woven mats derived from post-industrial textile scrap for thermal-acoustic dashboard dampeners and under-carpet insulation mats [3,15]. Concurrently, marine-derived biopolymers such as chitin and chitosan extracted from crustacean exoskeletons offer rigid, film-forming polysaccharide chains used as natural flame retardants and anti-microbial interior coatings [7]. Microalgae-derived bioplastics and algae-blended polyurethanes provide bio-content for flexible seating foams, reducing the automotive interior sector’s cradle-to-gate carbon footprint [1,15].

When benchmarking bioinspired composites against conventional metallic automotive baselines, distinct trade-offs emerge between lightweighting efficiency and environmental impact (Table 3). Continuous flax-reinforced bio-epoxy laminates achieve specific stiffness values (22.2–29.6 GPa g^−1^cm^3^) competitive with aluminum alloys (25.6 GPa g^−1^cm^3^) and high-strength steels (26.8 GPa g^−1^cm^3^) at a fraction of the cradle-to-gate global warming potential (1.8–2.4 vs. 8.5–11.2 CO_2_-eq kg^−1^ for aluminum). For dynamic crash applications, the literature results consistently agree that nacre-inspired laminates and functionally graded bamboo cellular structures generate stable, progressive crushing modes, achieving SEA ranges of 35–48 kJ kg^−1^ and 30–45 kJ kg^−1^, respectively exceeding stamped steel crash boxes (20–30 kJ kg^−1^). However, reported properties diverge significantly under varying testing conditions: while metallic alloys maintain consistent energy dissipation across multi-axial crash vectors, bio-composite SEA values drop by up to 35% when subjected to off-axis shear or high hygrothermal aging, highlighting that architectural fiber alignment must strictly follow primary impact trajectories.

### 3.3. Bioinspired Surface Engineering and Functional Coatings

Surface textures and coatings that reproduce biological surfaces are used to improve durability, reduce friction and drag, and add self-cleaning functions to automotive components. Unlike conventional coatings that primarily provide protection, bioinspired surfaces offer multiple functionalities, including corrosion resistance, friction reduction, self-cleaning, anti-fouling, drag reduction, and enhanced optical performance. Recent advances in micro-/nanofabrication, laser surface texturing, plasma processing, and additive manufacturing have enabled the development of multifunctional coatings inspired by natural systems such as lotus leaves, shark skin, and gecko feet.

(a) Lotus-Inspired Superhydrophobic Surfaces: In natural lotus leaves, the micron-scale papillae (10–20 μm) exceed the wavelength of visible light (λ ≈ 380–750 nm), generating intense diffuse light scattering that renders pristine biological surfaces opaque. To adapt the Cassie–Baxter mechanism (Section 2.2.3) for transparent automotive optical subsystems, engineered coatings decouple topography from optical scattering by synthesizing sub-wavelength nanotextures (<100 nm) using laser texturing, plasma etching, or nano-silica deposition. By matching the effective refractive index, these nanotextured surfaces preserve the Cassie–Baxter superhydrophobic state while achieving high visible light transmittance (>90%), providing durable self-cleaning, anti-icing, and anti-fouling functionality for ADAS camera lenses, LiDAR sensor covers, windshields, rearview mirrors, and solar roofs.
(b) Bioinspired Anticorrosive Barrier Coatings: Metallic automotive components (e.g., Al–Si–Cu die-cast powertrain parts and magnesium subframes) require surface protection against galvanic corrosion and chloride-induced pitting. Engineered coatings inspired by the leaf-cuticle wax layer and mussel-adhesive catechol chemistries provide dual-action protection: (i) dense crosslinked hydrophobic barrier networks that suppress water/electrolyte diffusion, and (ii) sacrificial microcapsule interlayers that release film-forming corrosion inhibitors upon microcrack initiation. Electrochemical impedance spectroscopy (EIS) benchmarks confirm that bioinspired self-healing epoxy coatings loaded with inhibitor-filled nanocontainers maintain low corrosion current densities (I_corr_ < 10^−8^ A cm^−2^) and low-frequency impedance moduli (|Z|_0.01 Hz_ > 10^8^ Ω cm^2^) following 1000 h of standard ASTM B117 salt-spray exposure [24], preventing underfilm corrosion creep across machined aluminum alloy interfaces [24,25].
(c) Shark Skin-Inspired Drag-Reducing Surfaces: Shark skin possesses aligned riblet microstructures that reduce turbulent flow and suppress vortex formation, thereby minimizing hydrodynamic drag. Biomimetic riblet surfaces fabricated through laser surface texturing and advanced coating technologies have demonstrated reduced aerodynamic drag, lower friction, improved lubricant retention, and enhanced corrosion resistance. These characteristics make them attractive for automotive underbody panels, heat exchangers, cooling channels, rotating shafts, and transmission components, where improved fluid flow and reduced energy losses enhance overall vehicle efficiency [24,26].
(d) Gecko-Inspired Dry Adhesive Surfaces: Translating the fibrillar contact mechanics established in Section 2.2.3, researchers have developed reusable dry adhesive systems for automotive electronic sensors, detachable interior panels, flexible electronics, robotic assembly, and wearable vehicle monitoring devices. These bioinspired adhesive surfaces facilitate easy assembly and disassembly while improving reliability, reducing residue, and supporting lightweight modular automotive designs [12,13,16].

### 3.4. Self-Healing and Smart Bioinspired Materials

Self-healing and smart materials inspired by living systems are one of the most promising developments in sustainable automotive engineering, allowing for autonomous repair of micro-scale damage to enable the extension of the service life of components, increase reliability and bring down maintenance costs. These materials have been designed to emulate the ability of biological tissues to heal if they become damaged, thus stopping microcracks from growing to a catastrophic failure. Based on the healing mechanism, self-healing materials can be divided into two categories: extrinsic and intrinsic, and they have different benefits for the automotive industry [27,28].

(a) Extrinsic Self-Healing Systems: These are materials that include healing agents in microcapsules or hollow fibres or interconnected vascular network within the material. When a crack is formed, these reservoirs break open and pour out healing agents, including dicyclopentadiene (DCPD), epoxy monomers, precursors for polyurethane, or linseed oil, into the damaged area to polymerize and repair the structure. This has been reported to achieve even better than 90% healing efficiencies and is thus very effective for repairing limited areas of damage in coatings and composite structures.
(b) Intrinsic Self-Healing Systems: Intrinsic self-healing systems are based on reversible chemical interactions of the polymer network without the need of stored healing agents. Self-repair is achieved by hydrogen bonding, ionic interaction, Diels-Alder reactions, disulfide exchange, and transesterification, which can be repeated without the loss of active ingredients. The materials are more durable, provide multiple healing cycles, and have greater long-term reliability than those made outside the body.
(c) Smart Sensing and Autonomous Damping in Automotive Systems: Integrated piezoresistive carbon/MXene sensor networks enable real-time structural health monitoring (SHM) by tracking crack propagation and impedance shifts under cyclic mechanical fatigue.

### 3.5. Bioinspired Thermal and Tribological Materials

In powertrain contacts (e.g., cylinder liners, piston skirts, camshaft lobes, and journal bearings), frictional dissipation accounts for up to 15–20% of total vehicle energy losses. Bioinspired surface texturing mimics the micro-grooved topology of shark skin (denticles) and the scale overlap of snake ventral scales. Laser-textured micro-dimple arrays and directional riblets (10–50 μm width, 5–15 μm depth) act as micro-hydrodynamic bearings and wear-debris traps under boundary and mixed lubrication regimes. In pin-on-disk and ring-on-liner tribometer trials under PAO/synthetic engine oil lubrication, textured surfaces achieve a 22–38% reduction in the friction coefficient (μ = 0.04–0.07 vs. 0.09–0.12 for untextured steel) alongside a >40% reduction in abrasive wear scar volume [23,26,29]. However, wear performance drops under boundary dry-sliding or severe abrasive contaminant ingress, where micro-cavities clog, transitioning the contact from hydrodynamic lift to accelerated three-body abrasive wear.

(a) Bioinspired Thermal Management Systems: Natural systems like leaves of a tree and termite mounds are also well-known to have extremely effective branching channel networks which carry maximum heat transfer with minimum pressure drops. Researchers have created biomimetic coolant channels by additive manufacturing systems to enhance coolant distribution in thermal management systems and electric vehicle battery packs, inspired by these designs. These optimized flow networks have been proven to lower the maximum battery cell temperature by around 5–10 °C than a conventional straight channel (no bypass), which improves battery safety, efficiency and service life.
(b) Applications of automotive engineering: Integrating these bioinspired flow architectures and surface textures mitigates parasitic mechanical dissipation and suppresses localized thermal hotspots. Primary automotive deployment targets include battery thermal cold plates, radiator heat exchangers, and high-load powertrain tribological interfaces (Table 4).

The wide range of sustainable materials found in contemporary vehicle systems illustrates their versatility in delivering multi-functional surface characteristics, lightweight performance, durability and crashworthiness in addition to thermal management. Table 4 attempts to summarize how some of the bioinspired materials are being integrated into important subsystems of an automotive system, matching the biological inspiration to its engineering analogues and main functional benefits [29,30].

**Table 4:** Automotive subsystem application matrix of bioinspired sustainable materials, their biological inspiration, engineering analogues, and functional benefits.

Automotive Subsystem	Conventional Material/Baseline System	Bioinspired Sustainable Solution	Biological Archetype	Primary Functional and Quantitative Benefits	References
Front Crash Box and Bumper Assembly	Stamped steel/6xxx-Al box sections	Continuous flax/bio-epoxy laminate with functionally graded cellular lattice core	Cortical bone and bamboo	40–50% mass reduction; progressive crush stability (SEA = 35–48 kJ kg^−1^) without brittle fragmentation	[1,6,10,14]
EV Battery Enclosure and Protection Trays	Welded stamped aluminium/sheet molding compound (SMC)	Bioinspired hybrid sandwich panel with bio-epoxy facesheets and hierarchical core	Glass sponge (Euplectella) and trabecular bone	High specific stiffness (E/ρ > 20 GPa g^−1^cm^3^); enhanced lateral puncture intrusion resistance; thermal barrier	[7,9,14]
Interior Consoles and Instrument Panel Trim	Mineral-filled polypropylene (PP/ABS)	Injection-molded bio-PP/PLA reinforced with micronized nutshell/fruit pomace fillers (10–40 wt%)	Hard endocarp sclerenchyma cell aggregates	Replaces mined talc; improves flexural modulus; eliminates sink marks; reduces cradle-to-gate GWP	[1,3,11,15]
Acoustic Floor Underlays and Cavity Mats	Petroleum polyurethane (PU) and EPS foams	Thermally deactivated mycelium foam grown on agricultural crop waste	Fungal hyphal network	Low density (0.10–0.35 g cm^−3^); high sound absorption coefficient (α > 0.7 at 1–4 kHz); char-forming flame retardancy	[1,3,11,15]
ADAS Sensors, LiDAR Covers and Glazing	Borosilicate glass/polycarbonate	Sub-wavelength (<100 nm) nanotextured superhydrophobic hybrid coatings	Lotus leaf	Cassie–Baxter self-cleaning (θ > 150°); >90% visible light transmittance; anti-icing and anti-fouling	[4,24,25]
EV Battery Thermal Management Channels	Extruded straight-channel aluminum cold plates	3D-printed hierarchically branched cooling flow channels	Leaf venation and termite mounds	Uniform coolant velocity; lower pressure drop; reduces peak battery pack temperature by 5–10 °C	[1,4,16]
Powertrain Tribological Interfaces (piston skirts, bearings, liners)	Honed cast iron/steel contacts	Laser surface-textured micro-dimple/riblet arrays	Shark skin denticles and snake scales	20–40% reduction in hydrodynamic friction coefficient (μ); enhanced lubricant retention; reduced wear scar volume	[12,23,26,29]
Exterior Body Panels and Clear Coats	Conventional polyurethane/acrylic coatings	Intrinsic/extrinsic self-healing bio-resins incorporating dynamic covalent bonds or microcapsules	Biological tissue repair	Autonomous microcrack healing (>90% recovery); suppresses moisture ingress; extended corrosion protection	[24,27,28,30]
Modular Interior/Sensor Mounting	Chemical adhesives and single-use mechanical clips	Micro/nanofibrillar dry-adhesive polymer patches	Gecko foot spatulae	Strong reversible shear adhesion via van der Waals forces; residue-free disassembly; modular circular assembly	[7,12,13]

## 4. Processing, Characterization, Sustainability Assessment and Performance Evaluation

The successful development of bioinspired automotive materials requires more than the replication of biological structures. It involves the integration of bioinspired design, sustainable material selection, scalable manufacturing, performance validation, and life-cycle considerations. Natural fibre composites, bio-based polymers, cellular structures, and hierarchical architectures are particularly sensitive to processing parameters such as temperature, pressure, moisture content, fibre orientation, porosity, and fibre–matrix interfacial bonding. Consequently, manufacturing strategies must simultaneously preserve the structural features responsible for bioinspired performance while minimizing material consumption, processing energy, waste generation, and environmental impacts. Recent advances in additive manufacturing, liquid composite molding, RTM, freeze casting, and digitally assisted manufacturing have enabled the fabrication of lightweight structures with controlled geometry, hierarchical architecture, and multifunctional properties [19,20,21,31,32,33]. Mechanical characterization and LCA should be performed on the same component to simultaneously verify structural compliance and quantify net environmental gains over conventional automotive baselines.

### 4.1. Advanced Manufacturing Technologies

Manufacturing technologies capable of reproducing natural architectures while maintaining the integrity of bio-based constituents are essential for producing bioinspired automotive components. Many lignocellulosic fibres undergo thermal degradation within relatively moderate temperature ranges; therefore, processing conditions must be carefully controlled to prevent fibre degradation and loss of mechanical performance. Low-temperature processing, controlled fibre alignment, near-net-shape manufacturing, and optimized material deposition can reduce both manufacturing defects and material waste.

#### 4.1.1. Additive Manufacturing

Additive manufacturing (AM) is one of the most promising approaches for fabricating bioinspired structures because of its ability to produce complex geometries with controlled internal architecture. Lattice structures inspired by bamboo, trabecular bone, honeycomb structures, and nacre can be manufactured using techniques such as fused filament fabrication, direct ink writing, selective laser sintering, binder jetting, and continuous-fibre 3D printing. An important sustainability advantage of AM is its ability to deposit material selectively rather than producing a component from a larger feedstock followed by extensive subtractive machining. Consequently, topology optimization can be combined with AM to reduce unnecessary material while retaining the required stiffness, strength, and energy-absorption characteristics. The approach is particularly relevant to lightweight automotive structures, where bioinspired cellular and lattice architectures can provide high specific mechanical performance with reduced material usage [19,31]. From a manufacturing standpoint, achieving competitive automotive build rates requires optimizing layer deposition thickness, print-bed thermal uniformity, and continuous-tow nozzle feed rates to minimize inter-layer voids without sacrificing dimensional fidelity [31].

#### 4.1.2. Liquid Composite Molding and Resin Transfer Molding

For larger structural components such as door panels, hoods, floor structures, body panels, and battery enclosures, liquid composite molding (LCM), vacuum-assisted resin infusion (VARI), and RTM provide scalable manufacturing routes for natural-fibre composites. In these processes, pre-arranged fibre preforms such as flax, hemp, kenaf, or other plant fibres are impregnated with polymeric resin under controlled pressure or vacuum. Appropriate process control can improve fibre wetting, reduce void formation, and enhance dimensional consistency. Compared with some conventional manufacturing routes, near-net-shape composite processing can reduce material waste and secondary machining. To align with high-volume automotive production takt times (<60 s), processing developments prioritize HP-RTM paired with rapid in-mold curing kinetics (100–120 °C) and automated preform compaction, ensuring complete fiber wetting without inducing thermal degradation in the lignocellulosic reinforcement.

#### 4.1.3. Freeze Casting and Bioinspired Cellular Fabrication

Freeze casting, or ice templating, is an emerging approach for producing anisotropic porous materials inspired by structures such as nacre, bone, wood, and other hierarchical biological materials. During directional freezing, growing ice crystals redistribute suspended particles or polymers, producing aligned architectures. Subsequent sublimation of the ice generates interconnected pores. Such architectures combine low density with high specific stiffness, crack deflection, and thermal barrier capability, making them promising for vehicle impact-energy management and battery cell thermal isolation [20,21,33]. Industrial translation of this architecture depends on regulating cold-finger temperature gradients and slurry solid loading to precisely tune pore aspect ratios, alongside developing continuous sublimation setups that accelerate ice removal.

### 4.2. Performance Characterization: A Concise Validation Framework

The characterization of bioinspired automotive materials remains necessary, but detailed descriptions of standard mechanical, thermal, microstructural, and environmental testing procedures are outside the principal scope of this review. Instead, characterization should be viewed as a performance-validation stage that establishes whether the proposed sustainable material can provide equivalent or improved functionality compared with conventional materials. Key characterization categories and their relevance to automotive applications are summarized in Table 5.

### 4.3. Sustainability Assessment and Life-Cycle Analysis

LCA provides a standardized, empirical methodology for benchmarking bioinspired materials against conventional automotive baselines [2,18]. Conducted in accordance with ISO 14040 and ISO 14044 standards, LCA systematically models goal and scope definition, life-cycle inventory (LCI), life-cycle impact assessment (LCIA), and interpretation [2,18].

**Figure 5:**
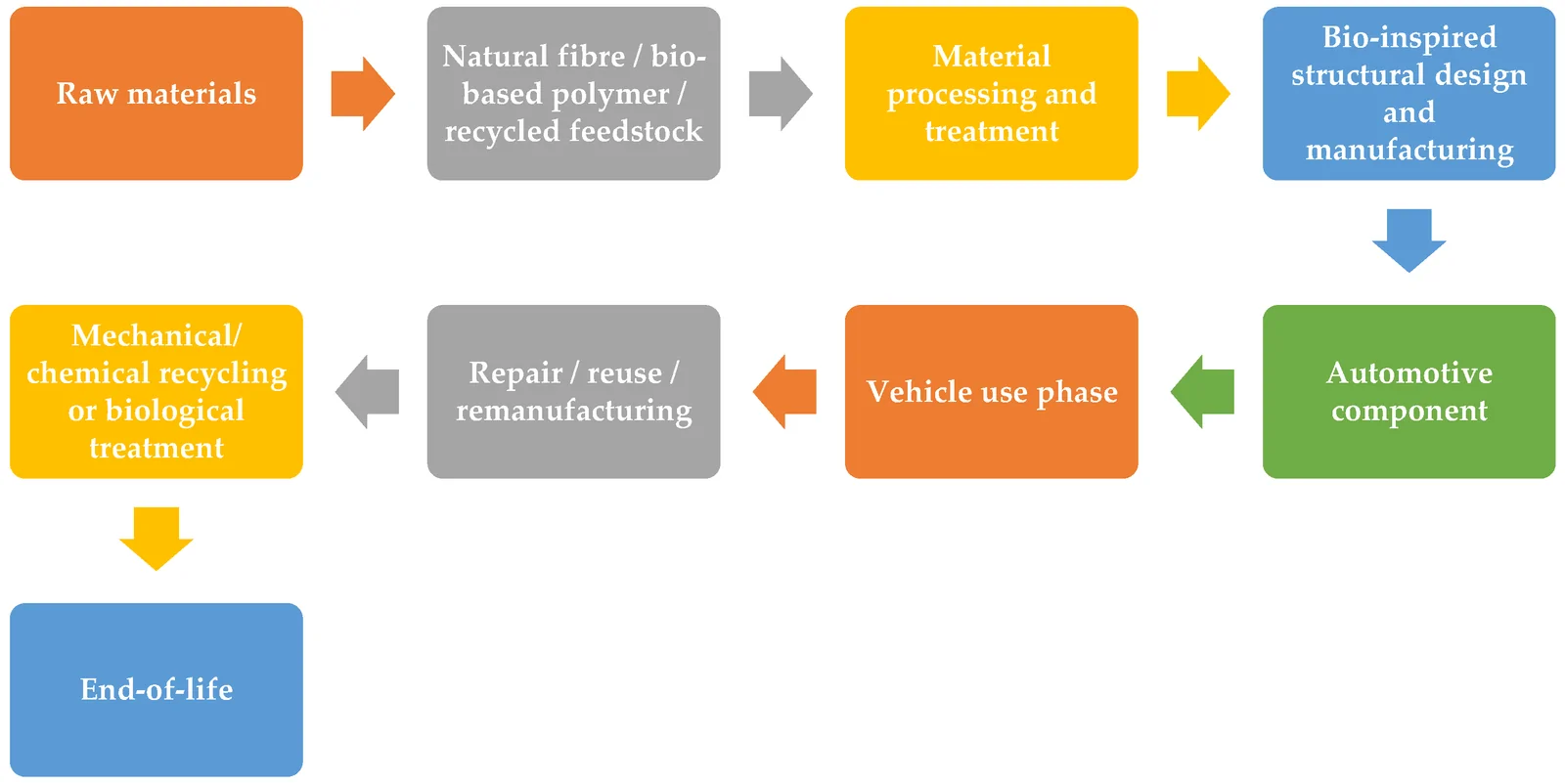
Schematic for life-cycle assessment of bioinspired automotive materials.

As illustrated in Figure 5, the life cycle of bioinspired automotive materials encompasses all stages from renewable or bio-based feedstock extraction, material processing, and bioinspired structural design to component manufacturing, vehicle use, maintenance, reuse or recycling, and end-of-life management. At each stage, key environmental indicators, including material and energy consumption, water usage, greenhouse gas emissions, waste generation, and transportation impacts, should be systematically evaluated. The functional unit should be defined by equivalent structural or service performance (e.g., one automotive component delivering a specified crash-energy absorption or torsional rigidity over a 150,000 km vehicle service life) rather than solely on gravimetric material mass, as simple mass comparisons (such as 1 kg of flax composite vs. 1 kg of glass-fiber composite) fail to capture differences in mechanical efficiency, lightweighting potential, and component durability [10,18].

#### 4.3.1. Environmental Impact Indicators

Several indicators are relevant for evaluating the environmental sustainability and functional performance of bioinspired automotive materials (Table 6). These metrics encompass environmental impacts, resource efficiency, service-life durability, and closed-loop restorative metrics such as the Material Circularity Index (MCI, an indicator measuring the degree to which linear material flows are minimized in favor of circular loops). By integrating these indicators within the defined functional unit and system boundary, comparative assessments establish transparent benchmarks against petroleum-derived and metallic automotive systems [2,18].

#### 4.3.2. Embodied Energy and Carbon Footprint

Natural-fibre composites can potentially reduce the environmental burden associated with conventional reinforcement materials because plant fibres are derived from renewable biological resources and generally require less energy-intensive processing than many mineral or synthetic reinforcements. However, net lifecycle performance depends heavily on cultivation inputs, surface treatment chemistry, processing temperature, and matrix selection. Consequently, carbon footprint reductions must be quantified through comparative cradle-to-grave accounting under standardized system boundaries rather than assumed as universal percentage gains [18].

#### 4.3.3. Lightweighting and Use-Phase Benefits

The sustainability assessment of automotive materials should not be restricted to the manufacturing stage. Mass reduction can influence vehicle energy consumption throughout the use phase, although the magnitude of this benefit depends on vehicle type, powertrain, driving conditions, component location, lifetime mileage, and the energy source. Architected cellular cores and high-specific-stiffness laminates enable structural mass reductions without compromising rigidity [8,10,20,31]. For electric vehicles, the corresponding use-phase assessment should consider vehicle mass, electricity consumption, battery size, electricity mix, and vehicle lifetime rather than assuming that a lighter component automatically produces a fixed carbon saving.

#### 4.3.4. Circularity, Recycling and End-of-Life

Circularity represents another major consideration for bioinspired automotive materials. While permanent covalent cross-linking historically constrained composite recovery, emerging dynamic covalent chemistries and bio-based thermoplastic matrices offer viable routes for closed-loop lifecycle integration. Previous research demonstrated heat- or water-driven malleability in a highly recyclable covalent network polymer, illustrating how reversible polymer-network chemistry can contribute to improved material recovery [40]. Similarly, research on self-healing polymers has demonstrated approaches that may extend component service life and reduce replacement-related material consumption [41,42]. Consequently, circularity assessments should directly utilize the quantitative closed-loop recovery and retention metrics outlined in Table 6 to evaluate net environmental benefits across successive vehicle life cycles.

#### 4.3.5. Sustainability Trade-Offs and Limitations

Despite their potential, bioinspired materials should not automatically be classified as environmentally superior. Several trade-offs may reduce or even eliminate their sustainability advantage. First, agricultural feedstock cultivation introduces direct environmental burdens through synthetic fertilizer synthesis, pesticide runoff, and freshwater consumption [18]. Second, thermochemical retting, fiber extraction, and silane/alkali surface modifications increase cumulative processing energy and chemical waste streams [23,43]. Third, combining bio-based reinforcements with non-recyclable petroleum thermosets or incompatible functional nanofillers obstructs closed-loop end-of-life recovery [40,44]. To resolve these conflicts, empirical cradle-to-grave LCA models must balance agricultural and chemical burdens against operational lightweighting gains across the full vehicle lifespan [18,45].

### 4.4. Integrated Performance–Sustainability Evaluation

The preceding discussion indicates that the evaluation of bioinspired automotive materials should integrate mechanical performance, durability, environmental impact, and circularity rather than treating these characteristics independently (Table 7).

## 5. Industrial Challenges, Commercial Case Studies, and Future Perspectives

### 5.1. Manufacturing Scale-Up and Economic Feasibility

One of the principal barriers to commercialization is translating laboratory-scale manufacturing techniques into high-volume automotive production. Many bioinspired architectures, including nacre-inspired laminates, bamboo-inspired functionally graded structures, trabecular bone lattices, and honeycomb cellular materials, are currently fabricated using additive manufacturing, freeze casting, layer-by-layer assembly, or controlled infiltration. Although these techniques provide excellent control over hierarchical architectures, their relatively slow production rates and high processing costs limit industrial scalability [40,43,45,46].

High-volume automotive manufacturing requires takt times typically below 60 s per part for injection-molded interior components and approximately 3–5 min for structural HP-RTM. In contrast, laboratory-scale bioinspired fabrication methods, particularly freeze casting and extrusion-based Direct Ink Writing (DIW), involve prolonged deposition, freezing, and sublimation stages extending from several hours to days. This substantial difference in production rate represents an important economic barrier to mass production. Industrial implementation requires reducing production cycles to meet automotive takt times. Automated fiber placement (AFP), reactive thermoplastic stamping, and high-pressure resin transfer molding (HP-RTM) can reduce cycle times to under 3–5 min for structural composite preforms. Furthermore, integrating inline near-infrared (NIR) or dielectric spectroscopic monitoring provides real-time tracking of resin flow front progression and degree of cure (α(t)), minimizing cycle overshoot and void formation during high-throughput processing [40,43,45,46].

Modern automotive manufacturing also requires high dimensional consistency, process repeatability, and stringent quality control. Current additive manufacturing technologies, including DIW, FFF, and stereolithography, require substantial improvements in deposition rate, automation, dimensional accuracy, and inline inspection before becoming economically competitive for large-scale automotive production.

Material variability represents another major challenge. Unlike metallic feedstocks, natural fibres exhibit variations in cellulose content, fibre diameter, crystallinity, moisture content, and tensile strength depending on geographical origin, cultivation practices, harvesting season, and extraction techniques. These variations directly influence composite performance; therefore, incoming fibre batches should be tested for diameter distribution, moisture content, and tensile scatter across the supply chain [40,45]. Furthermore, although raw agricultural fibres are relatively inexpensive, secondary processing, including fibre retting, chemical surface treatments, thermal drying, precise tow alignment, resin impregnation, and non-destructive inspection substantially escalates net component fabrication costs [43,45,46]. Establishing automated feedstock screening, standardized surface functionalization protocols, and lean preform consolidation will therefore be essential to lowering manufacturing costs while maintaining structural performance [43,45,46].

### 5.2. Long-Term Durability and Reliability

Automotive components are expected to operate reliably for more than fifteen years under cyclic mechanical loading and harsh environmental conditions. Consequently, understanding the long-term degradation behaviour of bio-based composites remains a major research priority.

#### 5.2.1. Moisture Degradation

Moisture diffusion into natural-fiber automotive composites during environmental exposure is commonly modeled using one-dimensional Fickian diffusion:

(4) $$\frac{\partial C}{\partial t}=D_{z}\frac{\partial^{2}C}{\partial z^{2}}$$

where C is moisture concentration, t is exposure time, and D_z_ is the through-thickness diffusion coefficient. For flax- and hemp-reinforced bio-epoxy composites immersed at room temperature (23 °C), typical measured diffusion coefficients range from D_z_ = 1.2 × 10^−6^ to 3.8 × 10^−6^ mm^2^ s^−1^. At saturation (M_∞_ ≈ 6–11 wt% water uptake), this diffusion process degrades fiber–matrix interfacial shear strength by up to 30–45%, explaining why hydrophobic barrier treatments are necessary for vehicle exterior panels [40,43,46].

#### 5.2.2. Thermal Stability and Fire Resistance

Many commercially available bio-polymers soften between 80 and 120 °C and undergo thermal degradation above approximately 200 °C, limiting their use in under-hood environments and electric vehicle battery systems. Emerging materials such as lignin-modified bio-epoxies, bio-benzoxazines, furan resins, vitrimers, ceramic-coated natural fibres, and phosphorus-containing flame retardants have demonstrated significant improvements in glass transition temperature, flame resistance, thermal conductivity, and dimensional stability while maintaining environmental sustainability [40,45].

#### 5.2.3. Fatigue Behaviour

Unlike metallic alloys that predominantly fail through single-crack propagation, hierarchical bio-composites accumulate damage across multiple constituent phases. The total damage parameter D can be expressed as a linear superposition:

(5) $$D=D_{f}+D_{m}+D_{i}$$

where D_f_, D_m_, and D_i_ represent normalized damage indices (0 ≤ D ≤ 1) corresponding to fiber fracture, matrix cracking, and interfacial debonding, respectively. In cyclic tension–tension fatigue testing (R = 0.1), micro-computed tomography (μCT) confirms that interfacial debonding (D_i_) initiates early in service life (N/N_f_ < 0.3), followed by progressive matrix microcracking (D_m_), while catastrophic fiber failure (D_f_) is restricted to the final 10% of fatigue cycles [41,42].

#### 5.2.4. Performance Envelopes, Contradictions, and Failure Envelopes in Bio-Composites

While laboratory studies report high ambient specific mechanical properties for bioinspired laminates, these systems exhibit defined operational limits where load-bearing performance drops sharply:

(a) Hygrothermal Degradation under prolonged humidity: Moisture diffusion along hydrophilic plant fibers leads to matrix plasticization and fiber swelling at >85% RH (50 °C), lowering interlaminar shear strength by 30–45% and reducing axial crash energy dissipation by up to 35% [23,35].
(b) Thermal softening near glass transition thresholds: Unmodified bio-based thermoplastics (PLA, PHA) exhibit low heat deflection temperatures (T_g_ ≈ 55–65 °C), causing matrix creep during cabin solar soak (>80 °C) and restricting structural use unless cross-linked with high-T_g_ vitrimers or bio-epoxies (T_g_ > 130 °C) [15,40].
(c) Anisotropic sensitivity to multiaxial loading: Because biological archetypes derive high stiffness along specific principal growth vectors, unoptimized laminates subjected to off-axis or transverse shear impact suffer premature delamination and longitudinal splitting, losing over 50% of their axial energy absorption capacity [10,23,40].

### 5.3. Circular Economy and End-of-Life Management

The automotive industry is rapidly transitioning toward circular manufacturing in response to increasingly stringent environmental regulations, including the European Green Deal, the Circular Economy Action Plan, and the End-of-Life Vehicle (ELV) Directive [44,45]. To overcome the recycling limitations of legacy cross-linked resins, bioinspired composites incorporating vitrimer-based dynamic networks and renewable matrices enable high-efficiency material reclamation [40,41]. Emerging recycling technologies including solvolysis, vitrimer reprocessing, enzymatic degradation, microwave-assisted depolymerization, and supercritical-fluid recycling enable the efficient recovery of intact reinforcing natural fibers and resin monomers without severe degradation of their mechanical properties [40,44,46].

A critical consideration in the lifecycle engineering of automotive biomaterials is the inherent paradox between operational durability and end-of-life biodegradability. Passenger vehicle components are strictly engineered to meet a service lifespan of 10 to 15 years, requiring high dimensional stability and resilience against severe environmental stressors, such as cyclic temperature fluctuations (–40 °C to 85 °C), ultraviolet (UV) radiation, prolonged moisture exposure, and contact with automotive fluids [43,47,48]. Consequently, rapid biodegradability under ambient environmental conditions while advantageous for single-use packaging is undesirable for functional automotive components, as uncontrolled degradation compromises crashworthiness and passenger safety [46,47]. To resolve this trade-off, automotive bio-composites often incorporate hydrophobic surface treatments, biostable bio-thermosets, or bio-based engineering thermoplastics (e.g., bio-polyamides, nutshell-filled polyolefins) that suppress biological degradation during vehicle service [40,48]. At the end of vehicle life, disposal strategies prioritize high-value circular pathways such as mechanical reprocessing, chemical depolymerization, and industrial composting in specialized facilities over uncontrolled environmental disposal [40,45].

LCA studies consistently demonstrate that natural-fiber-reinforced composites (such as flax, hemp, and kenaf) can achieve a 30–70% reduction in embodied carbon emissions compared with conventional glass-fiber-reinforced plastics (GFRPs) and mineral-filled petroleum polymers, owing to low embodied processing energy and biogenic carbon sequestration during crop growth [40,45,46]. The assessment should cover the whole life cycle: feedstock cultivation, processing energy, and end-of-life treatment [44,48]. Combining mass-efficient architectures with recyclable matrices addresses both the use-phase and the end-of-life impacts of automotive composites [49,50].

### 5.4. Data-Driven Design and Process Monitoring

Data-driven design frameworks integrate machine learning (ML) algorithms to optimize multi-variable composite configurations without exhaustive experimental trial-and-error. Machine-learning models can parameterize fiber orientation, fiber volume fraction (V_f_), stacking sequence, and cellular topology against objective functions including stiffness and specific energy absorption [44,45,48]. Specifically, Physics-Informed Neural Networks (PINNs) embed governing anisotropic elastoplasticity equations directly into loss functions, predicting non-linear progressive damage accumulation while reducing the number of high-fidelity finite element iterations [50,51,52,53].

In automated manufacturing cells, computer-vision algorithms paired with acoustic emission sensors monitor resin flow fronts and detect tow misalignment or void formation in real time during continuous filament placement [44,45,48].

Sensor-assisted process monitoring can improve manufacturing reliability in high-volume production. In liquid composite molding, embedded dielectric sensors and thermal imaging track resin flow fronts and cure progression, identifying unsaturated dry spots prior to complete cross-linking [44,45,48]. Similarly, integrating computer-vision systems with acoustic emission monitoring during continuous filament placement enables real-time detection of tow steering defects and void formation, ensuring geometric repeatability across structural automotive preforms [50,51,52,53].

### 5.5. Multifunctional and Bioinspired Composite Design

The development of multifunctional bioinspired composites is increasingly shifting from the optimization of individual properties toward the integration of multiple functions within a common material architecture. However, combining several functions in a single matrix or structural system introduces significant design trade-offs because modifications that improve one performance parameter can adversely affect another. For example, increasing reinforcement content or interfacial bonding to improve stiffness and load transfer may reduce ductility, damage tolerance, or processability, whereas introducing conductive phases for sensing or electromagnetic functionality can increase density and alter the matrix–reinforcement interface. Similarly, incorporating healing agents, functional coatings, or thermally active phases can interfere with structural continuity and long-term mechanical integrity [50,51,52].

A major challenge is therefore to maintain structural load-bearing capability while introducing additional functional constituents without creating preferential sites for crack initiation, interfacial debonding, moisture ingress, or thermal degradation. In protective composite systems, for example, the incorporation of functional surface layers must provide adequate adhesion to the substrate while remaining sufficiently stable under mechanical deformation and environmental exposure. Recent studies on bioinspired protective coatings for Al–Si–Cu alloys demonstrate the potential of combining self-healing and corrosion protection, but their practical implementation requires simultaneous consideration of coating adhesion, damage recovery, environmental stability, and compatibility with automotive manufacturing processes [53]. Likewise, bioinspired crashworthy structures require a balance between energy absorption and structural integrity, since excessive stiffness can increase peak transmitted loads whereas excessive compliance may compromise load-bearing capacity and intrusion resistance [54].

The integration of continuous-fibre reinforcement with biomimetic architectures presents a further challenge because hierarchical structural optimization must be compatible with fibre orientation, manufacturing constraints, and defect tolerance [55,56]. Changes in architecture intended to improve impact performance or mass efficiency may introduce local stress concentrations or complicate manufacturing and quality control [49,50,53]. Consequently, multifunctional engineering requires multi-objective optimization algorithms that balance structural load-bearing capacity against localized stress concentration factors and manufacturing cycle constraints [57,58].

The same design principle extends beyond automotive materials. Bioinspired architectures are being investigated for sustainable energy systems, environmental technologies, defence structures, aerospace components, and other lightweight engineering applications. These developments provide transferable approaches for controlling hierarchical structure, material interfaces, and multifunctional performance, while also highlighting the need to evaluate material sustainability across the complete life cycle [59,60,61,62,63]. For automotive subsystems, successful translation relies on mapping the physical interactions between active functional additives and primary load-bearing phases under real-world vehicle operating spectra.

### 5.6. Industrial Case Studies

Several automotive applications demonstrate the increasing use of natural-fibre composites and bioinspired architectures for lightweighting, functional improvement, and reduction in environmental impacts. To distinguish demonstrated industrial applications from emerging technologies, the following cases can also be considered according to their approximate Technology Readiness Level (TRL).

(a) Case Study 1: Porsche 718 Cayman GT4 Clubsport—TRL 8–9: Porsche has used flax-fibre composites with bio-based epoxy systems for body components such as doors, front splitters, rear wings, and engine covers. These components demonstrate the potential of natural-fibre composites to provide competitive stiffness and aerodynamic performance while reducing the environmental burden associated with conventional composite production. The application represents a relatively mature demonstration of natural-fibre composites in high-performance automotive structures [46].
(b) Case Study 2: BMW i3 and BMW iX—TRL 9: BMW has incorporated natural-fibre composites based on hemp, kenaf, and flax in components including door panels, dashboard carriers, seat backrests, and interior trim. Their low density and favourable acoustic and vibration-damping characteristics contribute to lightweighting and interior comfort, while renewable feedstocks can reduce the environmental impact of selected components [44,46,47].
(c) Case Study 3: Polestar Electric Vehicles—TRL 8: Polestar has investigated and implemented flax-fibre composite technologies incorporating PowerRibs™, a ribbed structural concept inspired by leaf-vein architectures. The rib geometry increases bending and torsional stiffness while reducing material consumption and component mass. The approach demonstrates how biological structural principles can be translated into lightweight automotive interior components [44,46,47,48].
(d) Case Study 4: Mercedes-Benz and Toyota—TRL 9: Mercedes-Benz and Toyota have used renewable-fibre-based materials, including kenaf, bamboo, and cellulose composites, in interior panels, parcel shelves, and trim components. These applications demonstrate the feasibility of incorporating bio-based reinforcement into automotive components while satisfying requirements for mechanical performance, durability, and occupant safety [39].
(e) Case Study 5: Electric Vehicle Battery Protection—TRL 4–5: Bone-inspired lattices, bamboo-inspired graded cellular structures, tubular lattices, and other architected materials manufactured using additive manufacturing have demonstrated potential for crash-energy absorption and lightweight protection of EV battery enclosures. These concepts can improve side-impact protection while reducing structural mass; however, most such architectures remain at laboratory or prototype-development stages and require further validation under realistic automotive crash conditions before large-scale commercialization [44,46,47,48,52,54,58].
(f) Case Study 6: Multifunctional Automotive Coatings—TRL 4–6: Bioinspired self-healing and anticorrosive coatings for aluminium die-cast automotive components represent an emerging multifunctional application. Such coatings combine corrosion protection with damage-recovery capability, potentially extending component service life and reducing maintenance requirements. However, further work is required to establish long-term environmental durability, coating adhesion, repair efficiency, and compatibility with industrial automotive coating processes [57].

### 5.7. Future Research Directions

Commercial adoption of bioinspired composites requires resolving specific physical trade-offs among interfacial adhesion, hygrothermal stability, and cycle time. Future investigations must prioritize quantitative evaluations linking microstructural mechanics to manufacturing scalability and environmental performance across the full component lifecycle.

To address interfacial weaknesses and parasitic mass penalties, bio-derived nanomaterials such as cellulose nanocrystals (CNCs), cellulose nanofibrils, and chitin nanofibers can be incorporated to reinforce fiber–matrix interfacial shear strength and reduce moisture permeability [49,50,51,55,56,60,62]. In parallel, hybrid reinforcement configurations that interleave natural fibers with basalt fibers, recycled carbon fibers, continuous-tow architectures, graphene, or MXenes can mitigate transverse brittleness, enhance multi-axial crashworthiness, and extend cyclic fatigue life while minimizing parasitic mass [60,61,63,64,65,66].

Advanced architected metamaterials and digital manufacturing represent another critical frontier. Biomimetic lattices, functionally graded honeycombs, and programmable cellular cores fabricated via multi-material additive manufacturing enable spatially tuned stiffness and crushing behavior with minimized material consumption [8,14,64,65,66]. Integrating generative structural optimization directly with life-cycle indicators ensures that lightweighting gains are achieved without inducing adverse manufacturing energy burdens [64,65,66]. Furthermore, artificial intelligence (AI), PINNs, multiscale constitutive modeling, and automated inverse design frameworks can establish quantitative links among biological microstructures, processing parameters, and mechanical-environmental responses, thereby accelerating materials discovery and virtual qualification [67,68,69]. Future investigations of architected lattice and sandwich systems must couple computational optimization with empirical sustainability quantification rather than relying on assumed benefits of renewable origin. For instance, Song et al. [70] established a benchmark by executing a comprehensive LCA on 3D-printed bioinspired sandwich plates with wood-fibre-reinforced PLA, demonstrating that additive manufacturing electrical energy and post-processing significantly influence cradle-to-grave carbon parity. Future studies should adopt this lead and go further by: (i) defining a functional unit tied to a real automotive component (e.g., meeting specified crash-energy absorption or flexural stiffness over a defined vehicle service life), (ii) incorporating manufacturing electrical energy, tooling, and end-of-life recovery inside the system boundary, and (iii) benchmarking the printed bioinspired core directly against conventional honeycomb cores and conventional production routes so the environmental claim is measured rather than assumed. Finally, scalable industrial translation will depend on establishing high-rate manufacturing processes, standardized ASTM/ISO testing data cards, closed-loop vitrimer recycling networks, and verified carbon-negative production routes [40,41,44,45,68,70]. A concise synthesis of key technological barriers, intervention strategies, and future automotive outlooks is summarized in Table 8.

## 6. Conclusions

This review has examined how three mechanisms found in biological structures—multi-scale load transfer, energy dissipation, and functional surfaces—have been applied to automotive components. Quantitative synthesis of published data establishes that nacre-like continuous flax/bio-epoxy laminates and functionally graded bamboo cellular lattices achieve SEA values between 35 and 48 kJ kg^−1^ under axial compression, compared to 20–30 kJ kg^−1^ for conventional stamped DP600 steel. Concurrently, cradle-to-gate global warming potential is reduced from 8.5 to 11.2 kg CO_2_-eq kg^−1^ for primary aluminum to 1.8–2.4 kg CO_2_-eq kg^−1^ for bio-based laminates.

However, technical boundaries govern in-service vehicle integration: elevated relative humidity (>85% RH) reduces interlaminar shear strength by 30–45%, matrix softening occurs in bio-thermoplastics above 60 °C, and off-axis impact resistance remains highly sensitive to fiber orientation. Resolving these boundaries requires coupling high-rate consolidation methods (HP-RTM, AFP) with high-T_g_ vitrimer chemistries and validating environmental gains through ISO 14040/14044-standardized cradle-to-grave lifecycle inventories. Further progress depends on (i) design tools that optimize stiffness, hygrothermal durability, and cycle time simultaneously—such as topology optimization supported by physics-informed neural networks—and (ii) recyclable matrix chemistries such as vitrimers.

## Figures and Tables

**Table 1:** Comparison of conventional automotive materials and bioinspired sustainable materials with their biological inspirations and representative applications.

Material Category	Representative Material System	Key Advantages	Major Limitations	Target Automotive Systems	References
High-Strength Steels (AHSS/UHSS)	DP600, Boron steel (22MnB5)	High energy dissipation, mature stamping lines, complete recyclability	High density (7.85 g cm^−3^), severe vehicle mass penalty	Safety cage, B-pillars, structural chassis	[1,2]
Lightweight Aluminum Alloys	5xxx/6xxx sheet, 7xxx extrusions	High strength-to-weight ratio, excellent natural corrosion resistance	High primary smelting energy (8.5–11.2 kg CO_2_-eq kg^−1^)	Hoods, suspension subframes, battery trays	[1,2]
Conventional Engineering Plastics	PP, ABS, PA66, Polycarbonate	Low material cost, rapid injection cycle times (<60 s)	Fossil feedstock reliance, landfill persistence, microplastic generation	Interior consoles, ducting, front grilles	[1,11]
Bioinspired Sustainable Composites	Continuous flax/bio-epoxy, bamboo lattices, mycelium cores	Low density (0.15–1.4 g cm^−3^), reduced embodied carbon, acoustic damping	Hygrothermal sensitivity, matrix softening (Tg < 120 °C), raw fiber property scatter	Crash boxes, battery packs, acoustic panels, interior trim	[1,3,10]

**Table 2:** Biological archetypes, deformation mechanisms, governing analytical parameters, and synthetic engineering analogues of bioinspired sustainable materials.

Biological Archetype	Key Microstructural Architecture	Dominant Deformation/Functional Mechanism	Governing Analytical/Quantitative Parameter	Synthetic Engineering Analogue	References
Nacre (Mother-of-Pearl)	Staggered inorganic platelets (~95 vol.%) in thin organic matrix	Interfacial shear sliding, mineral bridge pull-out, crack deflection	Effective aspect ratio: S_eff_ = l/t	Layered continuous flax/bio-epoxy prepregs, alumina/polymer scaffolds	[5,6,7,11]
Bamboo Culm	Radial functionally graded distribution of vascular bundles	Stress redistribution, localized buckling resistance, progressive collapse	Radial modulus gradient: E(r) = E_0_(r/R)^n^	Functionally graded cellular tubes, variable-density TPMS cores	[8,9,14]
Cortical Bone	Concentric osteon lamellae surrounded by compliant cement lines	Crack twisting, microcrack arrest, viscoelastic ligament bridging	Fracture toughness enhancement: K_IC_	Multi-scale continuous fiber architectures, co-extruded filaments	[6,7,10]
Lotus Leaf	Hierarchical micro-papillae (10–20 μm) coated with epicuticular wax	Cassie–Baxter stable air-pocket trapping, droplet roll-off	Apparent contact angle: cosθ_CB_ = f_1_ cosθ−f_2_	Sub-wavelength laser-textured nanotextures (<100 nm)	[4,12]
Shark Skin	Aligned longitudinal micro-riblets with interlocking denticles	Vortex lifting, turbulent boundary-layer drag reduction	Dimensionless groove spacing: s^+^ = (s · u_τ_)/ν	Laser-textured micro-grooved polymer films	[7,12]
Gecko Setae	Hierarchical branching fibrillar spatulae (200 nm tips)	Reversible dry adhesion via van der Waals interactions	Interfacial work of adhesion: W_adh_	Synthetic elastomeric micropillars (PDMS/bio-PU)	[12,13]

**Table 3:** Mechanical performance of representative lightweight bioinspired structural materials used in automotive engineering.

Material System	Biological Archetype	Primary Processing Route	Density (g cm^−3^)	Specific Stiffness (E/ρ, GPa g^−1^cm^3^)	SEA (kJ kg^−1^)	Global Warming Potential (kg CO_2_-eq kg^−1^)	Performance Agreement and Operational Limits	References
High-Strength Steel (DP600)	Conventional Metallic Benchmark	Stamping/Cold Forming	7.85	26.8	20–30	2.6–3.2	Isotropic performance; high mass and embodied carbon penalty.	[1,2]
Aluminum Alloy 6061-T6	Conventional Metallic Benchmark	Extrusion/Die Casting	2.70	25.6	25–40	8.5–11.2	Standard crash baseline; high primary extraction energy.	[1,2]
Mycelium Foam Core	Fungal Hyphal Web	Biofabricated and Mold Curing	0.15–0.30	0.8–2.1	4–8	0.4–0.8	Consensus on acoustic damping (α > 0.7); non-load-bearing.	[1,3,11,15]
Flax/Bio-Epoxy Laminate	Nacre (layered platelets)	HP-RTM/Compression Molding	1.35	22.2–29.6	35–48	1.8–2.4	High consensus on axial crash energy absorption; diverges under moisture exposure (>85% RH).	[3,10,15]
Bamboo-Graded Lattice	Bamboo (radial vascular gradient)	Continuous Fiber AM/VARI	0.95–1.20	15.8–22.5	30–45	2.1–2.8	Agreement on buckling resistance; limited by inter-layer delamination under transverse shear.	[8,9,14]
3D-Printed Wood-PLA Core	Trabecular Bone/Hexagonal	Fused Filament Fabrication (FFF)	0.85–1.10	9.5–14.2	22–32	1.4–1.9	Consensus on mass reduction; printing energy must be included in system boundary.	[14,15]

**Table 5:** Performance characterization of bioinspired automotive materials.

Characterization Category	Representative Properties	Typical Techniques/Standards	Relevance to Automotive Applications	References
Mechanical	Tensile, compressive, flexural and shear strength	ASTM D3039, ISO 527, ASTM D6641, ASTM D790	Structural panels, body components, brackets	[6,19,21]
Impact/crashworthiness	Impact strength, energy absorption, SEA	ASTM D7136, ASTM D6110 and component-level impact testing	Crash absorbers, battery protection, crash boxes	[10,20,33,34]
Fatigue	Fatigue strength and damage accumulation	ASTM D3479 and relevant component-level procedures	Long-term structural durability	[7,34]
Dynamic/viscoelastic	Storage modulus, loss modulus and damping	DMA, ASTM D4065	NVH components, interior panels and housings	[1,3,21]
Thermal	Thermal stability, glass-transition temperature, degradation temperature	TGA, DSC, TMA	Engine compartments, battery systems and thermal-management components	[15,21]
Environmental durability	Moisture absorption, UV ageing, thermal/hygrothermal ageing	ASTM D570, ASTM G154 and accelerated ageing procedures	Exterior components and under-body applications	[23,35]
Surface functionality	Wettability, roughness and surface morphology	Contact-angle measurement, AFM and microscopy	Self-cleaning, anti-fouling and drag-reducing surfaces	[25,36,37,38]
Internal architecture	Porosity, fibre distribution, defects and crack development	SEM, X-ray μCT	Cellular structures, sandwich panels and AM components	[19,31,39]

**Table 6:** Principal sustainability indicators for bioinspired automotive materials.

Indicator	What It Measures	Relevance to Automotive Materials	References
Global Warming Potential (GWP)	Greenhouse-gas emissions expressed as CO_2_-equivalent	Determines cradle-to-grave climate benefit vs. fossil polymers/GFRP	[1,2,18]
Cumulative Energy Demand (CED)	Total non-renewable primary energy required throughout the life cycle	Quantifies energy savings during raw material extraction and low-T composite processing	[1,2,18]
Water Footprint	Direct and indirect freshwater consumption during agricultural cultivation and processing	Critical metric for comparing natural bast fibers (flax, hemp, jute) vs. synthetic fibers	[2,3,18]
Material Efficiency	Structural mass and resource quantity required to deliver a target performance level	Central to bioinspired lightweighting (e.g., cellular and lattice core mass reduction)	[2,8,10,14]
Recycled Content	Fraction of post-industrial or post-consumer recovered material incorporated into the part	Benchmarks circular utilization (e.g., recycled cotton/wool acoustic mats)	[1,3,15]
Recyclability and Recovery Yield	Mass fraction and quality of fibers/monomers recoverable at vehicle end-of-life	Governs closed-loop compliance under EU End-of-Life Vehicle (ELV) Directives	[40,41,42]
Waste Generation	Scraps, trimming losses, and non-recyclable solid residues across manufacturing and EOL	Measures process efficiency in near-net-shape RTM and additive manufacturing	[2,19,31]
Biogenic Carbon Sequestration	Atmospheric carbon retained within lignocellulosic and bio-based polymers during growth	Offsets supply-chain processing emissions during cradle-to-gate phase	[1,3,18]
MCI	Level of linear vs. restorative material flow retention	Core metric for closed-loop recycling assessment	[2,40,41]
Service Life Durability	Operational lifespan (years or mileage) under cyclic mechanical and environmental loads	Ensures environmental gains are not offset by premature hygrothermal degradation	[23,35,43]

**Table 7:** Recommended integrated evaluation framework.

Evaluation Dimension	Representative Metrics	Key Question
Structural performance	Strength, stiffness, SEA	Can the material perform the required function?
Lightweighting	Density, component mass, specific strength	Can material quantity be reduced?
Durability	Fatigue, moisture, UV and thermal stability	Will the component maintain performance throughout service?
Manufacturing	Energy, yield, waste and cycle time	Can the material be produced efficiently?
Environmental performance	GWP, CED, water consumption	Is the material environmentally advantageous?
Circularity	Closed-loop recovery, circularity index (MCI)	Can material value be retained after use?
Economic feasibility	Material and processing cost	Can the technology be industrialized?
Safety and regulation	Fire, toxicity, crashworthiness and standards	Is the material suitable for automotive qualification?

**Table 8:** Consolidated challenges, technological solutions, and future implementation outlook for bioinspired sustainable automotive materials.

Challenge Domain	Current Industrial Limitation	Proposed Technological Solution	Future Automotive Outlook	References
High-Throughput Manufacturing and Scalability	Low deposition rates in AM processes and long molding cycle times unsuited for automotive takt times (<60 s).	High-speed additive manufacturing, continuous-fiber 3D printing, automated HP-RTM/AFP, and inline spectroscopic cure monitoring.	High-volume production of semi-structural body panels, floor modules, and crash members.	[31,45,53,68]
Feedstock Consistency and Environmental Durability	Natural fiber property scatter, moisture-induced swelling, interfacial debonding, and matrix softening at elevated temperatures.	AI-assisted feedstock screening, nanocellulose/graphene moisture barriers, hydrophobic silane coupling, and high-T_g_ vitrimer bio-resins.	Certified feedstocks and long-term durability (>15 years) in exterior, under-body, and battery systems.	[15,23,37,43,64,65]
Multifunctional Design and Fire Safety	Combustibility of lignocellulosic constituents and trade-offs between load bearing, sensing, and self-healing integration.	Bio-based phosphorus flame retardants, char-forming additives, supramolecular healing networks, and hybrid nano-reinforcements.	Fire-safe structural battery enclosures, smart sensory trims, and self-repairing exterior panels.	[7,9,48,50,57,60,62]
Generative Design and Predictive Modeling	Highly anisotropic, multi-scale damage evolution requiring extensive and costly physical crash/fatigue testing.	PINNs, generative structural optimization, multiscale progressive damage mechanics, and high-strain-rate finite element simulations.	Accelerated virtual crashworthiness qualification and mass-optimized metamaterial cores.	[8,14,34,52,58,67,69]
Empirical LCA and Closed-Loop Circularity	Inconsistent LCA system boundaries, unmeasured processing energy, and irreversible cross-linking in thermoset matrices.	Cradle-to-grave LCA using component-level functional units, dynamic covalent vitrimer networks, and selective solvolysis recycling.	Verified low-carbon footprint parity against metallic/honeycomb baselines and closed-loop ELV compliance.	[1,2,18,40,41,44,60,70]

## Data Availability

No new data were created or analyzed in this study. Data sharing is not applicable to this article.
